# Supplementary material for: Phenotypic variation in dorsal fin morphology of coastal bottlenose dolphins (Tursiops truncatus) off Mexico
Source: PeerJ. 2017 Jun 13;5:e3415. doi: 10.7717/peerj.3415 (PMC5472037; doi:10.7717/peerj.3415)
Supplement: Appendix S1 — The procedure to reconstruct average dorsal fin contours from point measurements. [file peerj-05-3415-s003.pdf]

APPENDIX A

TRIGONOMETRIC RELATIONS AMONG MORPHOMETRIC INDICES FOR DORSAL FIN SHAPE

Median values were used as representative measures of the standardized indices from the dorsal fins at each studied site. However, the segment $\overline{AB}$ was fixed to 10 relative units (*i.e.*, length = 10 relative units) in order to scale all the measured dorsal fins. The remaining measurements were scaled using local median values to solve the equations derived from the trigonometrically related indices; note that local median values from each index (*i*) are represented by the correspondent constant $k_{(i)}$.

For $\overline{C30B} \,/\, \overline{AB} = k_{(1)}$,

$$\overline{C30B} = \overline{AB} * k_{(1)}$$

For $\overline{C20B} \,/\, \overline{AB} = k_{(2)}$,

$$\overline{C20B} = \overline{AB} * k_{(2)}$$

For $\overline{C10B} \,/\, \overline{AB} = k_{(3)}$,

$$\overline{C10B} = \overline{AB} * k_{(3)}$$

For $\overline{C5B} \,/\, \overline{AB} = k_{(4)}$,

$$\overline{C5B} = \overline{AB} * k_{(4)}$$

For $\overline{C30D} \,/\, \overline{AB} = k_{(5)}$,

$$\overline{C30D} = \overline{AB} * k_{(5)}$$

For $\overline{C20D} \,/\, \overline{AB} = k_{(6)}$,

$$\overline{C20D} = \overline{AB} * k_{(6)}$$

For $\overline{C10D} \,/\, \overline{AB} = k_{(7)}$,

$$\overline{C10D} = \overline{AB} * k_{(7)}$$

For $\overline{C5D} \,/\, \overline{AB} = k_{(8)}$,

$$\overline{C5D} = \overline{AB} * k_{(8)}$$

For $\overline{AO} \,/\, \overline{OB} = k_{(9)}$,

$$\overline{OB} = 1/\, k_{(9)}+1$$

$$\overline{AO} = k_{(9)}/k_{(9)}+1$$

For $\overline{C30O} \,/\, \overline{DO} = k_{(10)}$,

$$\overline{C30O} = \overline{OB}/\mathrm{Tan}(60)$$

$$\overline{DO} = \overline{C30O} \,/\, k_{(10)}$$

Furthermore, to compute the Cartesian coordinates of the median reference points for each measured dorsal fin, we used the fixed length of segment $\overline{AB}$ (*i.e.*, 10 relative units), and then solved equations of the former trigonometrically related ratios. Coordinates for the anterior insertion of the dorsal fin on the back of the dolphin (*i.e.*, point B) were fixed in x=10, y=1, and the x, y values for the remaining landmarks were computed according to the following equations (note that numbers in parenthesis represent decimal degrees):

For point A,

x value = - $\overline{AB}$ * Sin (60) + $\overline{C30B}$

y value = $\overline{AB}$ * Sin (30)

For point B,

x value = 10

y value = 1

For point C30,

x value = $\overline{C30B}$

y value = 1

For point C20,

x value = - $\overline{C20B}$ * Cos (10) + $\overline{C30B}$

y value = $\overline{C20B}$ * Sin (10)

For point C10,

x value = - $\overline{C10B}$ * Cos (20) + $\overline{C30B}$

y value = $\overline{C10B}$ * Sin (20)

For point C5,

x value = - $\overline{C5B}$ * Cos (25) + $\overline{C30B}$

y value = $\overline{C5B}$ * Sin (25)

For point D,

x value = $\overline{C30D}$ * Cos (60)

y value = $\overline{C30D}$ * Sin (60)

For point O,

x value = $\overline{OB}$ * Cos (30)

y value = $\overline{OB}$ * Sin (30)
